# Protein Source and Quality for Skeletal Muscle Anabolism in Young and Older Adults: A Systematic Review and Meta-Analysis

**DOI:** 10.1093/jn/nxab055

**Published:** 2021-04-13

**Authors:** Paul T Morgan, Dane O Harris, Ryan N Marshall, Jonathan I Quinlan, Sophie J Edwards, Sophie L Allen, Leigh Breen

**Affiliations:** School of Sport, Exercise and Rehabilitation Sciences, University of Birmingham, Birmingham, UK; School of Sport, Exercise and Rehabilitation Sciences, University of Birmingham, Birmingham, UK; School of Sport, Exercise and Rehabilitation Sciences, University of Birmingham, Birmingham, UK; School of Sport, Exercise and Rehabilitation Sciences, University of Birmingham, Birmingham, UK; National Institute for Health Research, Birmingham Biomedical Research Centre at University Hospitals Birmingham NHS Foundation Trust, Birmingham, UK; School of Sport, Exercise and Rehabilitation Sciences, University of Birmingham, Birmingham, UK; School of Sport, Exercise and Rehabilitation Sciences, University of Birmingham, Birmingham, UK; School of Sport, Exercise and Rehabilitation Sciences, University of Birmingham, Birmingham, UK; National Institute for Health Research, Birmingham Biomedical Research Centre at University Hospitals Birmingham NHS Foundation Trust, Birmingham, UK

**Keywords:** aging, lean body mass, muscle protein synthesis, protein, resistance exercise training, sarcopenia, strength

## Abstract

**Background:**

There is much debate regarding the source/quality of dietary proteins in supporting indices of skeletal muscle anabolism.

**Objective:**

We performed a systematic review and meta-analysis to determine the effect of protein source/quality on acute muscle protein synthesis (MPS) and changes in lean body mass (LBM) and strength, when combined with resistance exercise (RE).

**Methods:**

A systematic search of the literature was conducted to identify studies that compared the effects of ≥2 dose-matched, predominantly isolated protein sources of varying “quality.” Three separate models were employed as follows: *1*) protein feeding alone on MPS, *2*) protein feeding combined with a bout of RE on MPS, and *3*) protein feeding combined with longer-term resistance exercise training (RET) on LBM and strength. Further subgroup analyses were performed to compare the effects of protein source/quality between young and older adults. A total of 27 studies in young (18–35 y) and older (≥60 y) adults were included.

**Results:**

Analysis revealed an effect favoring higher-quality protein for postprandial MPS at rest [mean difference (MD): 0.014%/h; 95% CI: 0.006, 0.021; *P* < 0.001] and following RE (MD: 0.022%/h; 95% CI: 0.014, 0.030; *P* < 0.00001) in young (model 1: 0.016%/h; 95% CI: −0.004, 0.036; *P* = 0.12; model 2: 0.030%/h; 95% CI: 0.015, 0.045; *P* < 0.0001) and older (model 1: 0.012%/h; 95% CI: 0.006, 0.018; *P <* 0.001; model 2: 0.014%/h; 95% CI: 0.007, 0.021; *P* < 0.001) adults. However, although higher protein quality was associated with superior strength gains with RET [standardized mean difference (SMD): 0.24 kg; 95% CI: 0.02, 0.45; *P* = 0.03)], no effect was observed on changes to LBM (SMD: 0.05 kg; 95% CI: −0.16, 0.25; *P* = 0.65).

**Conclusions:**

The current review suggests that protein quality *may* provide a small but significant impact on indices of muscle protein anabolism in young and older adults. However, further research is warranted to elucidate the importance of protein source/quality on musculoskeletal aging, particularly in situations of low protein intake.

See corresponding commentary on page 1677.

## Introduction

The regulation of skeletal muscle is a complex process that hinges on the dynamic balance between muscle protein synthesis (MPS) and muscle protein breakdown (1–4). Although promoting skeletal muscle anabolism is important for general health and performance, maintaining skeletal muscle across the life span is particularly important given its multifaceted role in maintaining whole-body metabolic homeostasis and locomotory capabilities. Numerous studies have shown that resistance exercise training (RET) combined with the ingestion of sufficient dietary protein leads to greater hypertrophy in both young and older individuals than RET alone (5). However, although young individuals demonstrate a pronounced muscle anabolic response to these stimuli, a blunted response has been observed in older adults [termed *anabolic resistance* (6–8)], which reinforces recommendations for higher daily protein intakes to support muscle maintenance (≥1.2 g · kg^−1^ · day^−1^) (9, 10).

The acute MPS response and accompanying chronic adaptations of skeletal muscle to RET may be dependent not only on dose (10–13) and timing of protein intake around resistance exercise (RE) (14–18) but also the source or quality of protein (19–22). Systematic reviews and meta-analyses have been conducted on the effects of protein dose (i.e., 9) and timing (i.e., 14) but not protein source/quality, per se. The quality of a given protein is defined by a number of factors, including the amino acid (AA) content (particularly leucine), AA profile and AA bioavailability (i.e., digestibility) combined with protein and/or AA needs, and the digestion kinetics and delivery of AAs to biological tissues for protein synthesis (23). For example, it is generally accepted that most plant-based proteins exhibit lower digestibility (24, 25), contain an incomplete essential amino acid (EAA) profile (24, 25), and thus may not adequately support muscle anabolism compared with dose-matched animal-based proteins. Furthermore, different animal protein sources display divergent EAA profiles, rates of digestibility, and capacity for MPS stimulation (e.g., milk-derived whey compared with casein).

Despite a number of comparisons of the muscle anabolic properties (i.e., impacts on MPS, intramuscular signaling, muscle strength, muscle hypertrophy) between protein sources of differing quality, conflicting evidence exists on the importance of protein quality for postprandial MPS and enhancing RET-mediated gains in muscle mass and strength. Although previous reviews have discussed the effect of protein source/quality on muscle remodeling (i.e., 21, 22), there is a clear need for further extensive statistical analyses to understand whether higher-quality protein sources induce favorable changes in MPS, strength, and lean body mass (LBM). Consequently, the primary purpose of this review was to determine the effects of different, predominantly isolated, protein sources of varying *quality* when combined with RE on acute postprandial MPS (at rest and with exercise) and longer-term changes in strength and LBM. We conducted a systematic review and random-effects meta-analysis that was more inclusive in nature compared with previous narrative reviews to provide a contemporary evidence-based assessment on the role of protein source/quality on indices of muscle anabolism. We also undertook further subgroup analyses to determine the effect of protein source/quality between young (18–35 y) and older (≥60 y) individuals. We hypothesized that, when matched for dose, higher-quality protein sources would be associated with superior postprandial MPS and enhanced LBM and strength accretion with prolonged RET and that this magnitude of effect would be greater in older individuals.

## Methods

### Search strategy

A literature search was conducted on Ovid EMBASE (1974 to October 1, 2020) and MEDLINE (1946 to October 1, 2020). The following search terms were used: muscle protein synth*, muscle protein synthesis, MPS, fractional synth*, fractional synthetic rate, FSR, phenylalanine, postprandial, protein quality, protein, essential amino acids, EAA, essential amino, DIAAS, PDCAAS, milk, whey, casein, soy, rice, wheat, pea, egg, hypertrophy, strength, training, resistance, exercise. The search was limited by identifying human studies only. Boolean operators *and* and *or* were used to combine search terms. The search strategy is presented in **Supplemental Table 1**. Reference lists of reviews focusing on protein nutrition and skeletal muscle metabolism were also scanned, and additional studies were identified that were relevant to this topic.

### Eligibility criteria

#### Type of studies

**FIGURE 1:**
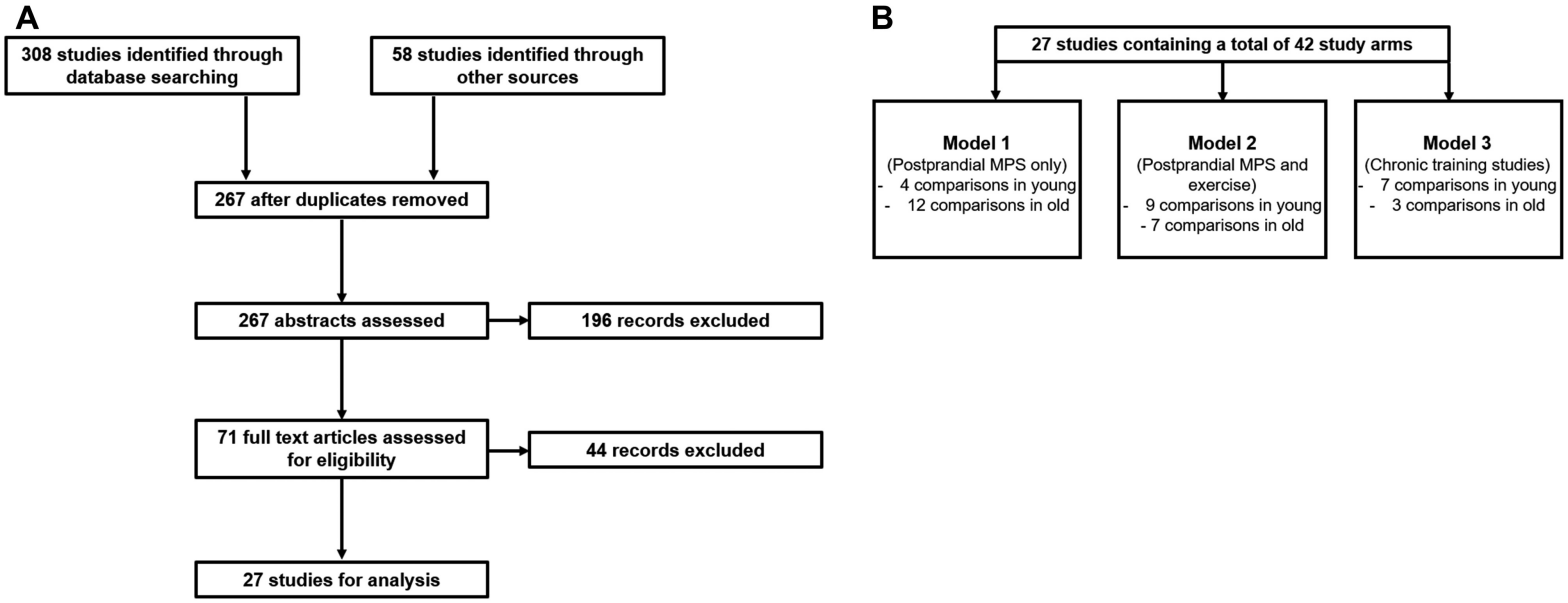
(A) Preferred Reporting Items for Systematic Reviews and Meta-Analyses flowchart for study selection. (B) A diagrammatic illustration of the 3 different models constructed for reporting evidence for/against lower- compared with higher-quality proteins. MPS, muscle protein synthesis.

Any study that included a comparison between ≥2 protein sources was considered eligible for inclusion. Both randomized and nonrandomized control trials were included for the acute postprandial MPS and RE-induced postprandial studies (models 1 and 2, respectively; see **Figure 1**A) as randomization is not always possible and/or these studies are not labeled as randomized control trials (RCT). All chronic protein-supplemented training studies (model 3) eligible for inclusion were required to be RCTs.

#### Participants

Studies that recruited healthy young or healthy older individuals were included. The mean age of the young individuals was set between 18 and 35 y, and the mean age for older participants was set at ≥60 y. This age limit was chosen as it has been reported that sarcopenia manifests during the fourth and fifth decade of life (26). As such, any study in which the mean age of participants was between 36 and 59 y was not included in the current review. Any other condition known to directly influence postprandial MPS, RE-induced MPS, or training adaptations was excluded to narrow the focus on the specific effect of protein source/quality on muscle remodeling.

#### Types of intervention

If a study compared ≥3 protein sources, the protein defined as the highest quality would then be compared, independently, with all other eligible proteins included within that study. Most studies included were of isolated supplemental protein sources due to the difficulty in directly assessing protein quality, per se, with whole foods as other nonprotein constituents may influence our primary outcome measures (27, 28). We decided to take a case-by-case semiquantitative approach to categorizing the protein quality of each protein source within each study as either high-quality (HIGH) or a lower-quality control (CON) protein. Protein quality was evaluated using a holistic approach of: total EAA content, leucine content, nonessential AA content, the limiting indispensable AA content, and, where possible, digestible indispensable amino acid score (i.e., DIAAS) to account for differences in digestibility. Protein dose was closely matched between protein sources. The final decision when categorizing a protein as either high or low quality was done qualitatively by 4 authors (DOH, RNM, PTM, and JIQ), considering all of the aforementioned combined with the postprandial time period of MPS assessment (for models 1 and 2) and the expected digestion and absorption characteristics. Where doses of specific AAs were not provided, estimates were computed based on information provided within each study and stated explicitly within the results (see **Tables 1** and **2**). Blends of proteins, AA-enriched supplements, and/or supplements with differential processing strategies were excluded unless a decision could be drawn to distinctively differentiate the protein quality between the experimental arms. For each study, the protein dose was considered either optimal (≥0.25 g · kg ^−1^, ∼20 g, or ≥1.6 g · kg^−1^ · day ^−1^ of protein for young and ≥0.4 g · kg ^−1^, ∼30 g, or ≥1.6 g · kg^−1 ·^day ^−1^ of protein for older) or suboptimal (<0.25 g · kg ^−1^, <20 g, or <1.6 g · kg^−1 ·^day ^−1^ of protein for young and <0.4 g · kg ^−1^, <30 g, or <1.6 g · kg^−1^ · day ^−1^ of protein for older) (9, 10). Although we acknowledge the potential influence of habitual dietary intake on postprandial MPS, the paucity reporting and/or controlling diet for models 1 and 2 meant that studies were not excluded based on habitual dietary intake. Given studies within these models assessed acute changes to MPS following an overnight fast, we would not expect habitual diet to have a marked effect on limiting AAs associated with a protein source and, therefore, a subsequent impact on our primary outcome measure. Although it appears that protein feeding prior to an experimental trial does not have a marked effect on the MPS response to subsequent feeding and/or exercise, we decided to exclude studies that did not enforce a fasted period prior to MPS assessment to reduce any potential confounding influence on our main outcome measure. For the acute exercise bout (i.e., model 2), only studies that used RE were included. Training programs (i.e., model 3) were only included if training was ≥6-wks as it has been shown that this is the minimum timeframe to display significant changes in LBM (29, 30). Studies for model 3 were also only included if there were ≥2 sessions per week, at least 50% of the training program incorporated resistance-based exercise (i.e., including concomitant training), and adherence to the training program was >85%.

#### Types of outcome measure

For the acute studies (i.e., models 1 and 2), the primary outcome measure was MPS. The assessment of postprandial MPS was set at a maximum of 8 h following the anabolic stimulus (i.e., feeding only) to account for differences in the absorption kinetics between fast- and slow-release proteins and within 24 h for model 2 (feeding plus RE), as muscle remains sensitive to anabolic stimuli, elevating MPS above baseline for an extended period of up to 24 h following the completion of RE (31). For studies where 2 protein boluses were consumed (i.e., 29), the assessment of MPS following the second bolus was not included to avoid any impact of a prior protein ingestion on the subsequent MPS response, in line with our fasted-state inclusion criteria. All studies in models 1 and 2 were required to have used the precursor-product model for calculation of fractional synthetic rate for in vivo MPS assessment in humans (32). Accordingly, any study using either a 2- or 3-pool arteriovenous model to assess MPS was excluded. The primary outcome measure for chronic training studies (i.e., model 3) was changes to LBM and/or strength. Studies were excluded if limb girth or skinfold calipers were used to determine LBM. Nevertheless, following a systematic review of the literature, of the 10 studies included within model 3, only 1 used hydrostatic body composition analysis (33), with the remaining studies using DXA to measure LBM (20, 34–41). Strength was assessed with 1–12 (or total) repetition maximum (RM), isokinetic, isometric, or isotonic strength. When there were several measures of the assessment of strength within 1 research study, the final decision for selecting the appropriate muscle group/action was completed by general consensus. Due to their relevance to locomotory function, compound lower body exercises and assessments of maximal strength (i.e., 1 RM, maximum voluntary contraction) were preferred.

### Data collection and analysis

#### Study selection

Figure 1 summarizes the study selection process. Assessment of titles and abstracts generated by the literature search was conducted by 2 independent reviewers (DOH and RNM). Any titles and abstracts that were deemed relevant to this review or potentially eligible for inclusion were kept, and the full-text articles were then obtained. The subsequent full-text assessment for eligibility was conducted by 2 independent reviewers (DOH and JIQ) (see Figure 1B). For all assessments, any disagreements between the 2 reviewers was resolved by consensus and, where necessary, a third and fourth reviewer (RNM and PTM).

#### Heterogeneity and risk of bias

Heterogeneity was assessed by χ^2^ and *I*^2^, with an α value of *P* < 0.05. Funnel plots that display trial treatment effect estimates against SE were visually inspected to determine publication bias in meta-analyses with ≥10 studies. However, no studies were removed based on publication bias assessment. A risk of bias assessment was completed using a domain-based questionnaire in line with the *Cochrane Handbook for Systematic Reviews of Interventions* (Chapter 8) using the following domains: randomization, allocation concealment, blinding of participants, blinding of outcome, incomplete data, selective reporting, and other bias. For each domain, a score was given as 1 (low risk), 0 (unclear risk), or –1 (high risk). This assessment was completed by 3 independent reviewers (DOH, RNM, and JIQ), and any disagreements were resolved by consensus. If a study had a score of 0, it was excluded from the meta-analysis (*n* = 0).

#### Data management

Three independent reviewers (DOH, SJE, and PTM) extracted all data from the studies deemed eligible for inclusion. All data were extracted on a study arm level to ensure that all relevant data obtained were used (i.e., if a study compared >2 eligible protein sources). Where relevant data were not reported, the corresponding author was contacted and a request submitted for additional data. If additional data were not acquired, an estimate was used that was completed by 2 independent reviewers (DOH and SJE) and/or was calculated using baseline values and/or percentage change, where appropriate, and verified by a third reviewer (PTM). Where it was not possible to obtain raw data from the study publication and/or contact with the authors, data extraction from tables or by interpolation from figures was completed using the Web Plot Digitizer (WebPlotDigitizer v4.3; Pacifica).

**TABLE 1:** Summary of the included studies in model 1 (effect of protein source/quality on postprandial muscle protein synthesis) and model 2 (effect of protein source/quality on resistance exercise–induced postprandial muscle protein synthesis) in young and older adults^1^

Study ID author, year	Study arm	Participants, *n*	Group, age, y	Body mass, kg	Sex, M/F	Total protein dose, g	EAA dose, g	Leucine content,^2^ g	Resistance exercise protocol (if applicable)	Main findings (postprandial MPS, fractional synthetic rate, %/h)
Bendtsen, 2019 (42)			Young							
	Hydrolyzed whey (HIGH)	18	24 ± 1	75 ± 2	M	15	6.2	1.9	—	NSD in Myofibrillar (MyoMPS) between whey (0.058 ± 0.007%/h) compared with either hydrolyzed porcine muscle (0.063 ± 0.011%/h) or hydrolyzed porcine blood (0.048 ± 0.007%/h) in healthy men. Postprandial period: 0–150
	Hydrolyzed porcine blood protein	18	24 ± 1	75 ± 2	M	15	6.1	1.7	—	
	Hydrolyzed porcine muscle protein	18	24 ± 1	75 ± 2	M	15	3.1	0.8	—	
Burd, 2012 (43)			Old	BMI^3^						
	Whey isolate (HIGH)	9	72 ± 1	26 ± 2	M	20	10.2	2.8	—	MyoMPS was significantly elevated (*P* < 0.05) with whey (0.043 ± 0.009%/h) compared with micellar casein (0.024 ± 0.005%/h) in healthy elderly men. Postprandial period: 0–240 min
	Micellar casein	7	72 ± 1	kg/m^2^	M	20	8.2	1.6	—	
Burd, 2012 (43)			Old	BMI^3^						
	Whey isolate (HIGH)	9	72 ± 1	26 ± 2	M	20	10.2	2.8	Unilateral knee extensions; 3 sets of 10 repetitions at each participant's 10RM	MyoMPS was significantly elevated (*P* < 0.05) with whey (0.055 ± 0.009%/h) compared with micellar casein (0.035 ± 0.005%/h) in healthy elderly men. Postprandial period: 0–240 min
	Micellar casein	7	72 ± 1	kg/m^2^	M	20	8.2	1.6		
Burd, 2015 (44)			Young							
	Skim milk (HIGH)	12	22 ± 1	74 ± 3	M	30	13.0	2.7	Leg press and knee extensions; 4 sets of 8–10 repetitions until volitional fatigue	NSD in MyoMPS between milk (0.071 ± 0.017) and minced beef (0.057 ± 0.021%/h) in healthy young men. Postprandial period: 0–300 min
	Minced beef	12	22 ± 1	74 ± 3	M	30	13.0	2.5		
Churchward-Venne, 2019 (45)			Young							
	Whey (HIGH)	12	23 ± 1	76 ± 2	M	20	10.1	2.6	Concurrent exercise bout including 4 sets of 8	NSD in MyoMPS between whey (0.054 ± 0.007%/h) compared with both micellar casein (0.059 ± 0.002%/h) or milk (0.059 ± 0.001%/h,
	Micellar casein	12	24 ± 1	73 ± 3	M	20	9.2	2.0		
	Milk	12	24 ± 1	74 ± 2	M	20	7.8	1.7		
									repetitions (∼80% 1RM) for both supine leg press and seated leg extensions followed by a 30-min continuous cycle (∼60% of maximal workload)	*P* > 0.05) in healthy, young, active men. Postprandial period: 0–360 min
Churchward-Venne, 2019 (46)			Young							
	Whey (HIGH)	12	23 ± 1	76 ± 2	M	20	10.1	2.6	Concurrent exercise bout including 4 sets of 8 repetitions (∼80% 1RM) for both supine leg press and seated leg extensions followed by a 30-min continuous cycle (∼60% of maximal workload)	NSD in MyoMPS between whey (0.054 ± 0.007%/h), soy (0.053 ± 0.013%/h, *P* > 0.05) in healthy, young, active men. Postprandial period: 0–360 min
	Soy	12	23 ± 1	74 ± 2	M	20	6.8	1.4		
Dideriksen, 2011 (47)			Old							
	Whey (HIGH)	6	64 ± 1	76 ± 4	M = 4; F = 2	15.6–30.4	∼12.3	∼3.4	Bilateral leg press and unilateral knee extensions; 5 sets of 8 repetitions at 80% 1RM	NSD in Myo and collagen MPS between whey (0.090 ± 0.012%/h) and caseinate (0.090 ± 0.007%/h, *P* > 0.05) in healthy elderly individuals. Postprandial period: 0–360 min
	Caseinate	6	70 ± 2	76 ± 6	M = 3; F = 3	(0.45 g/kg LBM)^4^	∼11.0	∼2.5		
Gorissen, 2016 (48)			Old							
	Whey (HIGH)	12	72 ± 2	79 ± 2	M	35	16.6	4.4	—	MyoMPS was significantly elevated (*P* < 0.05) with whey (0.041 ± 0.017%/h) compared with wheat (0.035 ± 0.017%/h) and wheat protein hydrolysate (0.032 ± 0.014%/h) in healthy older men. NSD difference was observed when whey was compared with micellar casein (0.050 ± 0.017%/h, *P* > 0.05). Postprandial period: 0–240 min
	Micellar casein	12	73 ± 1	75 ± 3	M	35	14.9	3.2	—	
	Wheat	12	68 ± 1	77 ± 2	M	35	9.8	2.5	—	
	Wheat protein hydrolysate	12	72 ± 2	79 ± 4	M	35	9.9	2.5	—	
Hamarsland, 2017 (49)			Young							
	Native whey (HIGH)	10	25 ± 2	70 ± 12	M = 5; F = 5	2 × 20 g^5^	10.6	2.7	Leg press and knee extensions; 4 sets of 8 repetitions to failure (i.e., 8RM)	MyoMPS was significantly elevated (*P* < 0.05) with native whey (0.110 ± 0.066%/h) compared with milk (0.081 ± 0.047%/h) protein in young resistance-trained men and women. Postprandial period: 60–180 min
	Milk	12	25 ± 5	73 ± 12	M = 8; F = 4	2 × 20 g^5^	9.3	2.0		
Hamarsland, 2019 (50)			Old							
	Native whey (HIGH)	11	73 ± 3	70 ± 12	M = 6; F = 5	2 × 20 g	10.6	2.7	Leg press and knee extensions; 4 sets of 8 repetitions to failure (i.e., 8RM)	MyoMPS was significantly elevated (*P* < 0.05) with native whey (0.119 ± 0.061%/h) compared with milk (0.068 ± 0.038%/h) protein in healthy elderly men and women. Postprandial period: 60–180 min
	Milk	10	75 ± 4	76 ± 18	M = 7; F = 3	2 × 20 g	9.3	2.0		
Oikawa, 2020 (51)			Old							
	Whey (HIGH)	10	67 ± 2	80 ± 13	F	30	15.4	4.3	—	MyoMPS was significantly elevated (*P* < 0.0001) with whey (0.035 ± 0.011%/h) compared with collagen (0.030 ± 0.011%/h) in healthy older women. Postprandial period: 0–240 min
	Collagen	10	69 ± 4	71 ± 17	F	30	5.6	0.9	—	
Oikawa, 2020 (51)			Old							
	Whey (HIGH)	10	67 ± 2	80 ± 13	F	30	15.4	4.3	Four sets of unilateral knee extension exercise (∼60% 1RM) of the dominant leg	MyoMPS was significantly elevated (*P* < 0.0001) with whey (0.051 ± 0.009%/h) compared with collagen (0.035 ± 0.009%/h) in healthy older women. Postprandial period: 0–240 min
	Collagen	10	69 ± 4	71 ± 17	F	30	5.6	0.9		
Pennings, 2011 (52)			Old							
	Whey (HIGH)	16	73 ± 1	76 ± 2	M	20	9.3	2.4	—	Mixed MPS was significantly elevated (*P* < 0.05) with whey (0.15 ± 0.08%/h) compared with both casein (0.08 ± 0.04%/h) and casein hydrolysate (0.10 ± 0.04%/h) in healthy older men. Postprandial period: 0–360
	Casein	16	74 ± 1	75 ± 3	M	20	7.9	1.4	—	
	Casein hydrolysate	16	74 ± 1	76 ± 2	M	20	7.9	1.4	—	
Reitelseder, 2011 (53)			Young							
	Whey isolate (HIGH)	9	28 ± 2	79 ± 3	M	17.5	8.8	2.1	Unilateral seated leg extension; 10 sets of 8 repetitions at 80% 1RM	NSD in MyoMPS between whey (0.123 ± 0.016%/h) and casein (0.098 ± 0.011%/h, *P* > 0.05) in healthy male adults. Postprandial period: 60–360 min
	Casein caseinate	9	28 ± 2	79 ± 3	M	17.5	8.1	1.5		
Reitelseder, 2019 (54)			Old							
	Caseinate MIPRODAN40 (HIGH)	9	68 ± 2	80 ± 3	M	∼38	17.9	3.6	—	NSD in MyoMPS between caseinate MIPRODAN40 (0.045 ± 0.009%/h) and whey PEPTIGEN IF-3090 (0.043 ± 0.013%/h, *P* > 0.05) in healthy older males. Postprandial period: 60–360 min
	Whey PEPTIGEN IF-3090	10	69 ± 2	85 ± 3	M	(0.45 g/kg LBM)^4^	17.8	3.3	—	
Reitelseder, 2019 (54)			Old							
	Caseinate MIPRODAN40 (HIGH)	9	68 ± 2	80 ± 3	M	∼38	17.9	3.6	Unilateral leg extensions; 10 sets of 8 repetitions at 70% 1RM	NSD in MyoMPS between caseinate MIPRODAN40 (0.043 ± 0.012%/h) and whey PEPTIGEN IF-3090 (0.041 ± 0.013%/h, *P* > 0.05) in healthy older males. Postprandial period: 60–360 min
	Whey PEPTIGEN IF-3090	10	69 ± 2	85 ± 3	M	(0.45 g/kg LBM)^4^	17.8	3.3		
Tang, 2009 (55)			Young							
	Whey hydrolysate (HIGH)	6	23 ± 4	87 ± 14	M	21.4	10.0	2.3	—	Mixed MPS was significantly elevated (*P* < 0.01) with whey hydrolysate (0.091 ± 0.006%/h) compared with micellar casein (0.047 ± 0.004%/h) in healthy young men. NSD was observed when comparing whey hydrolysate with soy (0.077 ± 0.014%/h, *P* > 0.05). Postprandial period: 0–180
	Micellar casein	6	23 ± 4	87 ± 14	M	21.9	10.1	1.8	—	
	Soy	6	23 ± 4	87 ± 14	M	22.2	10.1	1.8	—	
Tang, 2009 (55)			Young							
	Whey hydrolysate (HIGH)	6	23 ± 4	87 ± 14	M	21.4	10.0	2.3	Unilateral leg press and knee extensions; 4 sets at 10–12 RM	Mixed MPS was significantly elevated in whey hydrolysate (0.150 ± 0.13%/h) compared with micellar casein (0.069 ± 0.005%/h, *P* < 0.01) and soy (0.116 ± 0.010%/h, *P* < 0.05) in healthy young men. Postprandial period: 0–180
	Micellar casein	6	23 ± 4	87 ± 14	M	21.9	10.1	1.8		
	Soy	6	23 ± 4	87 ± 14	M	22.2	10.1	1.8		
Walrand, 2016 (56)			Old							
	Soluble milk protein (HIGH)	8	72 ± 1	70 ± 2	M	15	7.1	1.8	—	MyoMPS was significantly elevated with soluble milk protein (0.062 ± 0.025%/h) and casein (0.027 ± 0.032%/h, *P* < 0.05) in healthy elderly men. Given fractionally every 20 min. postprandial period: 0–240 min
	Casein	7	72 ± 1	76 ± 3	M	15	6.6	1.4	—	
Walrand, 2016 (56)			Old							
	Soluble milk protein (HIGH)	8	73 ± 1	70 ± 2	M	30	14.3	3.6	—	NSD in MyoMPS between soluble milk protein (0.053 ± 0.031%/h) and casein (0.050 ± 0.014%/h, *P* > 0.05) in healthy elderly men. Given fractionally every 20 min. postprandial period: 0–480 min
	Casein	8	72 ± 1	76 ± 3	M	30	13.2	2.8	—	
Wilkinson, 2007 (57)			Young							
	Skim milk (HIGH)	8	22 ± 0	82 ± 6	M	18.2	∼7.8	∼1.6	Unilateral leg press, hamstring curl, and knee extension. For each exercise, 4 sets at 80% 1RM (10 repetitions for first 3 sets and the last set to exhaustion)	Mixed MPS was significantly elevated (*P* < 0.05) with milk (0.10 ± 0.01%/h) compared with soy (0.07 ± 0.01%/h) protein. Postprandial period: 0–180 min
	Soy	8	22 ± 0	82 ± 6	M	18.2	∼7.5	∼1.3		
Yang, 2012 (58)			Old							
	Whey (HIGH)	10	72 ± 5	81 ± 9	M	20	9.2	2.0	—	MyoMPS was significantly elevated (*P* < 0.005) with whey (0.043 ± 0.009%/h) compared with soy protein (0.029 ± 0.007%/h) in older healthy men. Postprandial period: 0–240 min
	Soy	10	72 ± 6	78 ± 11	M	20	7.1	1.6	—	
Yang, 2012 (58)			Old							
	Whey (HIGH)	10	70 ± 4	81 ± 12	M	40	18.4	4.0	—	MyoMPS was significantly elevated (*P* < 0.005) with whey (0.055 ± 0.009%/h) compared with soy (0.035 ± 0.008%/h) protein in older healthy men. Postprandial period: 0–240 min
	Soy	10	70 ± 5	77 ± 9	M	40	14.2	3.2	—	
Yang, 2012 (58)			Old							
	Whey (HIGH)	10	72 ± 5	81 ± 9	M	20	9.2	2.0	Unilateral knee extensions involving 3 sets at 10RM	MyoMPS was significantly elevated (*P* < 0.001) with whey (0.055 ± 0.009%/h) compared with soy (0.041 ± 0.010%/h) protein in older healthy men. Postprandial period: 0–240 min
	Soy	10	72 ± 6	78 ± 11	M	20	7.1	1.6		
Yang, 2012 (58)			Old							
	Whey (HIGH)	10	70 ± 4	81 ± 12	M	40	18.4	4.0	Unilateral knee extensions involving 3 sets at 10RM	MyoMPS was significantly elevated (*P* < 0.001) with whey (0.082 ± 0.038%/h) compared with soy (0.056 ± 0.009%/h) protein in older healthy men. Postprandial period: 0–240 min
	Soy	10	70 ± 5	77 ± 9	M	40	14.2	3.2		

1 Values are means ± SDs. EAA, essential amino acid; MPS, muscle protein synthesis; Myo, myofibrillar; NR, not reported; NSD, no significant difference; RM, repetition maximum.

2 Where EAA and/or leucine content was not provided, total content is provided as estimates based on information provided within each study, where possible.

3 BMI reported in the absence of body mass.

4 Estimates based on range of doses provided relative to body mass.

5 Two separate doses of a 20-g dose of protein provided.

**TABLE 2:** Summary of the included studies in model 3 (effect of protein source/quality when combined with resistance exercise training on longer-term adaptations to lean body mass and strength) in young and older adults^1^

Study ID, author, y	Study arm	Participants, *n*	Body mass, kg	Group, age, y	Sex, M/F	Protein dose, g · d^−1^	EAA dose,^2^ g · d^−1^	Resistance exercise training protocol	Diet control	LBM	Muscle strength	Summary of main findings
Brown, 2004 (33)				Young								
	Whey	9	81 ± 3	20 ± 0	M	33	12.3	9-wk whole-body strength training incorporating 14 exercises; 3 sets of 4–6 repetitions	Diet record	↑	—	NSD between whey and soy in the relative change in LBM (+2.2 ± 2.1 vs. +1.7 ± 1.8%) in experienced male weightlifters
	Soy	9	79 ± 5	22 ± 0	M	33	7.2			↑	—	
Fabre, 2017 (34)				Young								
	100% fast protein [FP(100)]	10	74 ± 7	27 ± 6	M	20	6.1	9-wk whole-body resistance training. Participants trained 4 d per-wk and every 3-wks, the 1RM was increased from ∼60% 1RM to 85% 1RM with repetitions lowered	Yes + Diet record	↑	↑	NSD in LBM between FP(100) (pre: 58.9 ± 6.8 vs. post: 60.4 ± 7.0 kg) and FP(20) (pre: 62.0 ± 5.4 vs. post: 63.4 ± 5.0 kg) in recreationally resistance-trained men. NSD in squat 1RM between FP(100) (pre: 96 ± 26 vs. post: 111 ± 26 kg) and FP(20) (pre: 97 ± 17 vs. post: 111 ± 17 kg)
	20% fast protein [FP(20)]	10	78 ± 7	26 ± 5	M	20	5.2			↑	↑	
Gryson, 2014 (35)				Old								
	Leucine-rich protein (Prolacta)	8	84 ± 2	61 ± 1	M	10	5.2	16-wk, 3 sessions per-wk, concurrent endurance and resistance exercise training involving whole-body resistance training with load progressively increased throughout the training program	Diet record	↔	↑	NSD in LBM between Prolacta (pre: 60.1 ± 8.8 vs. post: 60.7 ± 8.2 kg) and PL (pre: 62.3 ± 6.3 vs. post: 63.1 ± 7.2) in older healthy men. NSD in MVC between Prolacta (pre: 647 ± 40 vs. post: 662 ± 51 N) and PL (pre: 632 ± 51 vs. post: 650 ± 63 N)
	Placebo milk drink (containing 4 g of total milk protein)	9	82 ± 1	61 ± 1	M	10	1.7			↔	↑	
Hamarsland, 2019 (36)				Old								
	Native whey	15	78 ± 16	73 ± 2	M = 9; F = 6	40	20.4	11-wk whole-body resistance training program; 3 sessions per-wk comprising loads between 6RM and 12RM	Diet record	↑	↑	NSD in LBM between native whey (pre: 49.5 ± 10.9 vs. post: 51.3 ± 10.9 kg) and milk (pre: 49.8 ± 9.2 vs. post: 52.1 ± 9.2 kg) in healthy, active elderly men and women. NSD in leg press 1RM between native whey (pre: 158 ± 50 vs. post: 212 ± 63 kg) and milk (pre: 176 ± 55 vs. post: 222 ± 56 kg)
	Milk	15	75 ± 14	74 ± 4	M = 9; F = 6	38.2	17.2			↑	↑	
Hamarsland, 2019 (37)				Young								
	Native whey	18	78 ± 12	29 ± 6	M = 10; F = 8	40	20.4	12-wk whole-body resistance training; 3 sessions per-wk comprising loads between 6RM and 12RM	Diet record	↑	↑	NSD in LBM between native whey (pre: 54.2 ± 8.0 vs. post: 57.2 ± 8.2 kg) and milk (pre: 53.2 ± 10.7 vs. post: 55.8 ± 11.4 kg) in young untrained individuals. NSD in leg press 1RM between native whey (pre: 269 ± 77 vs. post: 344 ± 83 kg) and milk (pre: 266 ± 80 vs. post: 343 ± 74 kg)
	Dried milk	18	78 ± 16	29 ± 6	M = 10; F = 8	38.2	17.2			↑	↑	
Hartman, 2007 (20)				Young								
	Milk	18	79 ± 3	18–30^3^	M	35	∼15.1	Whole-body resistance training for 12-wk, 3 sessions per-wk. Participants completed 3–4 sets with repetitions between 6 and 12 at 80% 1RM	Diet record	↑	↑	Milk demonstrated a significantly greater increase in DXA-measured fat- and bone-free mass (pre: 62.4 ± 1.7 vs. post: 66.3 ± 1.6 kg) compared with soy (pre: 64.0 ± 2.5 vs. post: 66.8 ± 2.5 kg) in young, novice, male weightlifters. NSD in incline leg press 1RM between milk (pre: 186 ± 11 vs. post: 377 ± 18 kg) and soy (pre: 213 ± 15 vs. post: 423 ± 32 kg)
	Soy	19	83 ± 4	18–30^3^	M	35	∼11.9			↑	↑	
Joy, 2013 (38)				Young								
	Whey isolate	12	76 ± 6	21 ± 2	M	48	25.1	8-wk whole-body resistance training consisting of 'hypertrophy' sessions (8–12RM) and strength sessions (2–5RM). Participants trained twice per-wk	Yes	↑	↑	NSD in LBM between whey isolate (pre: 59.6 ± 5.2 vs. post: 62.8 ± 5.2 kg) and rice isolate (pre: 58.5 ± 5.5 vs. post: 61.0 ± 5.6 kg) in young resistance-trained males. NSD in leg press 1RM between whey isolate (pre: 210 ± 35 vs. post: 290 ± 40 kg) and rice isolate (pre: 220 ± 39 vs. post: 287 ± 37 kg)
	Rice isolate	12	76 ± 6	21 ± 2	M	48	17.4			↑	↑	
Lynch, 2020 (39)				Young								
	Whey	26	67 ± 10	18–35^3^	M = 10; F = 16	19	9.2	Whole-body resistance training for 12-wk, 3 sessions per-wk. Training sessions varied between 60% and 80% 1RM	Diet record	↑	↑	NSD in LBM between whey (pre: 44.5 ± 8.7 vs. post: 46.0 ± 8.9 kg) and soy (pre: 44.1 ± 10.3 vs. post: 45.2 ± 10.3 kg) in untrained young men and women. NSD in peak knee flexion or extension torque between whey (pre: 124 ± 40 vs. post: 164 ± 40 N·m) and soy (pre: 132 ± 45 vs. post: 160 ± 44 N·m)
	Soy	22	66 ± 13	18–35^3^	M = 7; F = 15	26	9.5			↑	↑	
Thomson, 2016 (40)				Old								
	High dairy protein	54	79 ± 15	61 ± 7	M = 25; F = 29	27^4^	NR	Whole-body resistance training for 12-wk, 3 sessions per-wk; 4 sets per exercise varying from 8–12 repetitions	Yes	↑	↑	NSD in LBM between high dairy protein diet (pre: 49.6 ± 11.0 vs. post: 50.6 ± 11.2 kg) and high soy protein diet (pre: 49.4 ± 11.2 vs. post: 50.8 ± 11.2 kg) in healthy older adults. NSD in knee extensor MVC between high dairy protein diet (pre: 132 ± 55 vs. post: 158 ± 62 kg) and high soy protein diet (pre: 142 ± 62 vs. post: 161 ± 62 N·m).
	High soy protein	64	79 ± 13	62 ± 8	M = 29; F = 35	27^4^	NR			↑	↑	
												Significant difference in total body 8RM between high dairy protein (pre: 149 ± 51 vs. post: 280 ± 88 kg; change: 131 ± 54 kg) and high soy protein (pre: 169 ± 79 vs. post: 271 ± 122 kg; change: 102 ± 51 kg)
Wilborn, 2013 (41)				Young								
	Whey	8	66 ± 5	20 ± 2	F	48	∼21.3	8-wk periodized resistance training program consisting of 2 upper-body and 2 lower-body workouts per-wk (4 sessions per-wk). Sport-specific training was completed alongside (basketball)	Diet record	↑	↑	NSD between whey (2.3 ± 1.5%) and casein (2.1 ± 1.5%) in the relative change of LBM in trained female basketball players. Casein and whey supplement groups both experienced significant strength gains for both leg press 1RM (whey: 88.7 ± 43.9 kg; casein: 90.0 ± 48.5 kg) and bench press 1RM (whey: 7.5 ± 4.6 kg; casein: 4.3 ± 4.5 kg), but there was no significant between-group difference in strength gains
	Casein	8	68 ± 3	21 ± 3	F	48	∼20.3			↑	↑	

1 Values are means ± SDs. Arrows represent increase or decrease in lean body mass and/or strength. FP, fast protein; LBM, lean body mass; MPS, muscle protein synthesis; MVC, maximum voluntary contraction; NSD, no significant difference; NR, not reported; PL, placebo; RM, repetition maximum.

2 Where EAA content was not provided, total content is provided as estimates based on information provided within each study, where possible.

3 Age ranges provided in the absence of means ± SDs.

4 The 27-g protein supplement was provided in addition to high dairy/soy protein diet, respectively.

#### Method of data synthesis

For models 1 and 2, MPS is provided as absolute postprandial rates, as basal rates of MPS have been shown to not differ between a range of populations and interventions (59–63). Furthermore, basal rates of MPS were not always reported and/or not measured in selected studies. For model 3, the relative change in LBM and strength was calculated to account for potential differences between independent groups and measurements. All studies that reported the SEM were converted to SD. If a study only reported the baseline and preintervention values for LBM and strength, the relative change SD was calculated using a computed correlation coefficient (*corr*) and using the following equation according to the *Cochrane Handbook for Systematic Reviews of Interventions* (64):

(1) $$\begin{eqnarray*}
\Delta SD = \sqrt {S{D_{pre}}^2 + S{D_{post}}^2 - 2 \times corr \times S{D_{pre}} \times S{D_{post}}} .
\end{eqnarray*}$$

Data were uploaded into RevMan (Review Manager, v5.4; The Cochrane Collaboration). If a study compared ≥3 protein sources or had multiple study arms (i.e., rested and exercise limb in a unilateral limb model), then they were uploaded as separate studies to isolate the intervention of interest. Further, where included studies had multiple treatment arms, shared groups were split into ≥2 groups with smaller sample sizes to account for “double-counts,” as per the *Cochrane Handbook for Systematic Reviews of Interventions* (64). Although we acknowledge that, as the resulting comparisons remain correlated, this method only partially overcomes the unit-of-analysis error, this approach allows for the inclusion of multiple treatment arms within 1 study and is conservative in its estimates of effect (64). Further, due to the inclusion of both crossover and parallel trials in our analyses, we must acknowledge that treating crossover trials as if they are parallel trials contributes to unit-of-analysis error. However, this method of analysis is conservative, in that crossover studies are under- rather than overweighted (64).

#### Meta-analyses

A random-effects meta-analysis was employed for all main outcome measures. All meta-analyses were performed using RevMan (Review Manager, v5.4; The Cochrane Collaboration), and data are presented as the mean difference (MD) or standardized mean difference (SMD), the respective 95% CIs, and effect size. SMD was used for model 3 as LBM and strength were measured in a variety of ways. The MD or SMD represents the size of the difference between the 2 proteins supplemented within each study and its effect on postprandial MPS (model 1), RE-induced MPS (model 2), and the relative change in LBM and strength from pre- to postresistance training intervention (model 3). The SMD is calculated as follows (64):

(2) $$\begin{eqnarray*}
SMD = \frac{{Difference\,\,in\,\,mean\,\,outcome\,\,between\,\,groups}}{{Standard\,\,deviation\,\,of\,\,outcome\,\,among\,\,participants}}.
\end{eqnarray*}$$

Subgroup analysis was performed on all main outcomes in which the studies were separated based on either investigating young or older adults. An α value of 0.05 was set for statistical significance. In addition to the meta-analytical assessment of protein source/quality between young and older adults, a systematic qualitative assessment of the efficacy of protein source/quality between age groups was also completed due to low study numbers. Multiple sensitivity analyses were performed for each model by excluding studies one at a time to determine if any of the results were influenced by the studies that were removed.

## Results

### Participant characteristics

A summary of each of the studies is presented throughout Tables 1 and 2. The mean age for the young groups across all 3 models ranged between 20 and 29 y, and the older groups were between 61 and 75 y. Of the 27 studies included, 18 exclusively investigated males (20, 42, 43–46, 33, 52–58, 35, 38, 40) and 2 exclusively investigated females (51, 41), with the remainder assessing a mix of sexes (47, 49, 50, 36, 37, 39, 40). A total of 7 included studies explicitly investigated individuals with RET experience (i.e., ≥6 months prior to recruitment).

### Protein supplementation

Most studies included in this meta-analysis compared whey with casein, soy, and/or milk protein variations, with differences in the processing of each protein, including a mix of hydrolysates, isolates, and concentrates (see Tables 1 and 2 for summary). For model 1, the protein dose ranged from 15.0 to 40.0 g, which equated to 6.2–18.4 g and 3.1–17.8 g of EAAs for the HIGH and CON proteins, respectively. Total leucine content ranged from 1.8 to 4.4 g and 0.8 to 3.3 g, corresponding to HIGH and CON. As selected studies in model 2 defined the dose relative to lean body mass, the average dose was calculated based on participant characteristics provided in each study, ranging from protein doses of 17.5 to 40.0 g and EAA content of 7.8 to 18.4 g and 5.6 to 17.8 g for HIGH and CON, respectively. Total leucine content ranged from 1.6 to 4.3 g and 0.9 to 3.3 g for HIGH and CON, respectively. Protein dose for model 3 within the protein supplement ranged from 10.0 to 48.0 g, which equated to 5.2–25.1 g and 1.7–20.3 g of EAAs for HIGH and CON, respectively. Daily protein intake for model 3 was recorded at 1.5 ± 0.3 g · kg^−1^· day^−1^ (*n* = 7).

### Resistance exercise characteristics

A summary of the RET protocols can be seen within Tables 1 and 2 for models 2 and 3, respectively. For model 2, most studies used a single bout of RE involving either leg press and/or knee extensions. The total number of sets ranged from 3 to 10 per exercise (4.7 ± 2.3 sets), and repetitions ranged from ∼8 to 12 (8.9 ± 1.1 repetitions). Five studies used a unilateral limb model in which 1 leg was exercised and the other was rested and, in these cases, could then be included in both model 1 and model 2 (43, 51, 54, 55, 58). Two studies incorporated both a resistance and endurance exercise bout (i.e., concurrent), in which participants performed a 30-min continuous cycle following resistance exercise (45, 46). For model 3, the length of training ranged from 8- to 16-wk (11.0 ± 2.5 wk). All of the studies within this model involved whole-body resistance exercise. Sessions ranged from 3 to 4 per week (3.3 ± 0.5 sessions/wk). One study involved both whole-body resistance exercise and endurance training (35). Another study investigated collegiate female basketball players, which incorporated sport-specific training alongside a resistance training program (41).

### Meta-analysis

**FIGURE 2:**
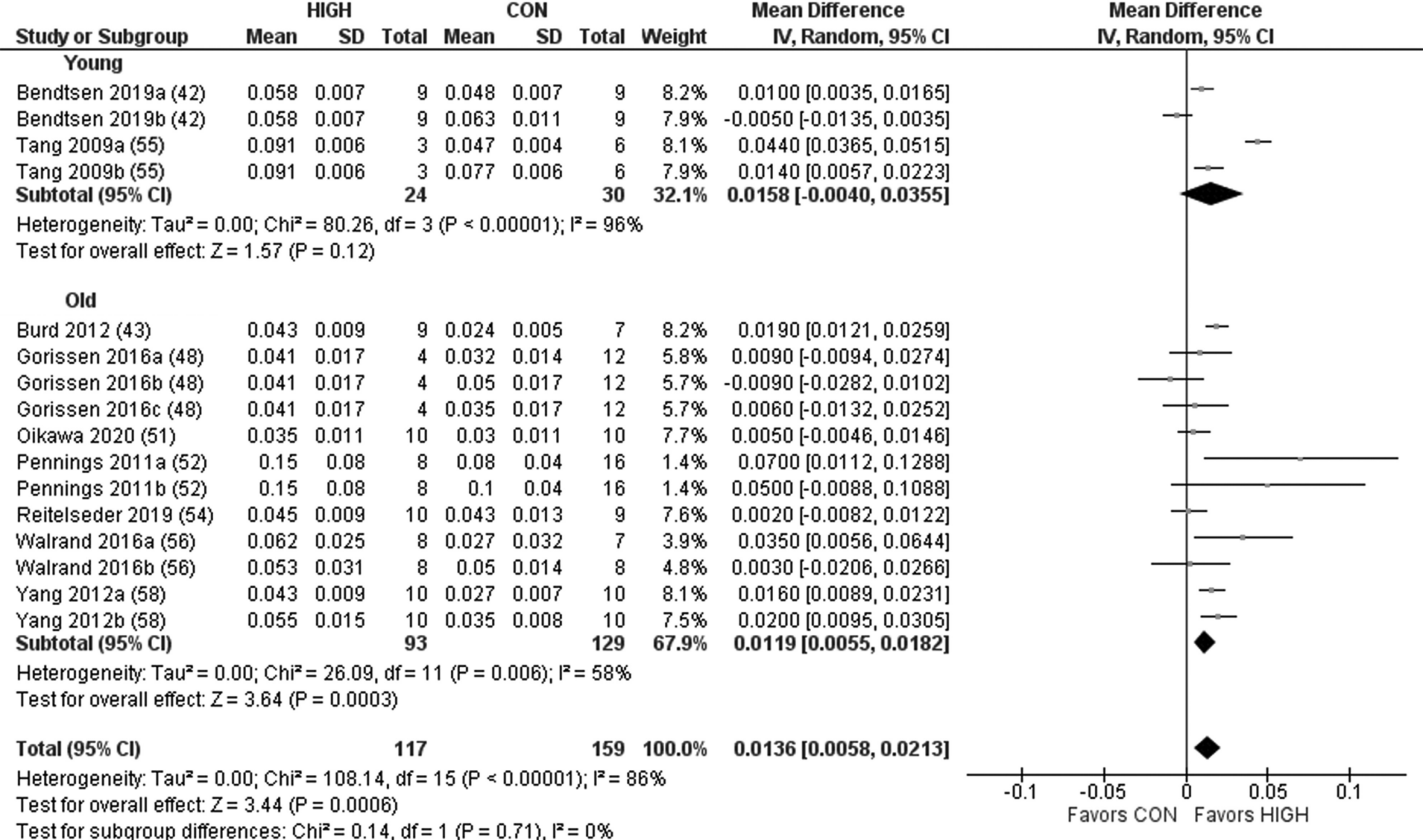
Forest plot of the results from a random-effects meta-analysis shown as the mean difference (MD) with 95% CIs on postprandial muscle protein synthesis rates in young and older participants (model 1). Values on x-axes represent MD. For each study, the symbol represents the standardized mean difference of the intervention effect with the horizontal line intersecting it as the lower and upper limits of the 95% CI. The vertical line represents nil effect of either a lower- or higher-quality protein source. The rhombi represent the weighted young, older, and total group's mean difference. The left-hand side favors CON and right-hand side favors HIGH. Where studies have multiple eligible treatment groups, the letters following the study year denote the separate study arm. CON, control protein; HIGH, high-quality protein.

**FIGURE 3:**
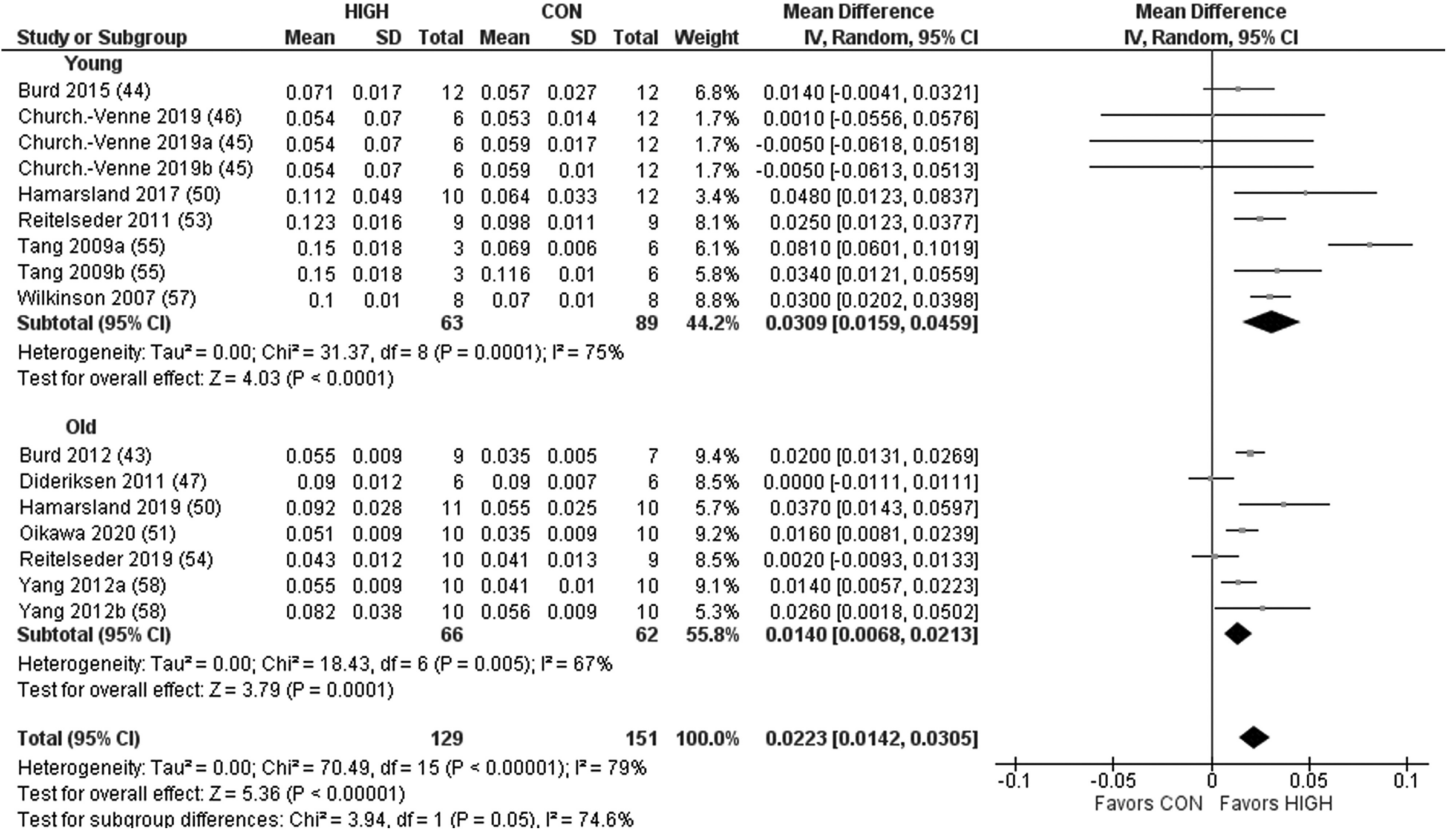
Forest plot of the results from a random-effects meta-analysis shown as the mean difference (MD) with 95% CIs on resistance exercise–induced postprandial muscle protein synthesis in young and older participants (model 2). Values on x-axes represent MD. For each study, the symbol represents the standardized mean difference of the intervention effect with the horizontal line intersecting it as the lower and upper limits of the 95% CI. The vertical line represents nil effect of either a lower- or higher-quality protein source. The rhombi represent the weighted young, older, and total group's mean difference. The left-hand side favors CON and right-hand side favors HIGH. Where studies have multiple eligible treatment groups, the letters following the study year denote the separate study arm. CON, control protein; HIGH, high-quality protein.

**FIGURE 4:**
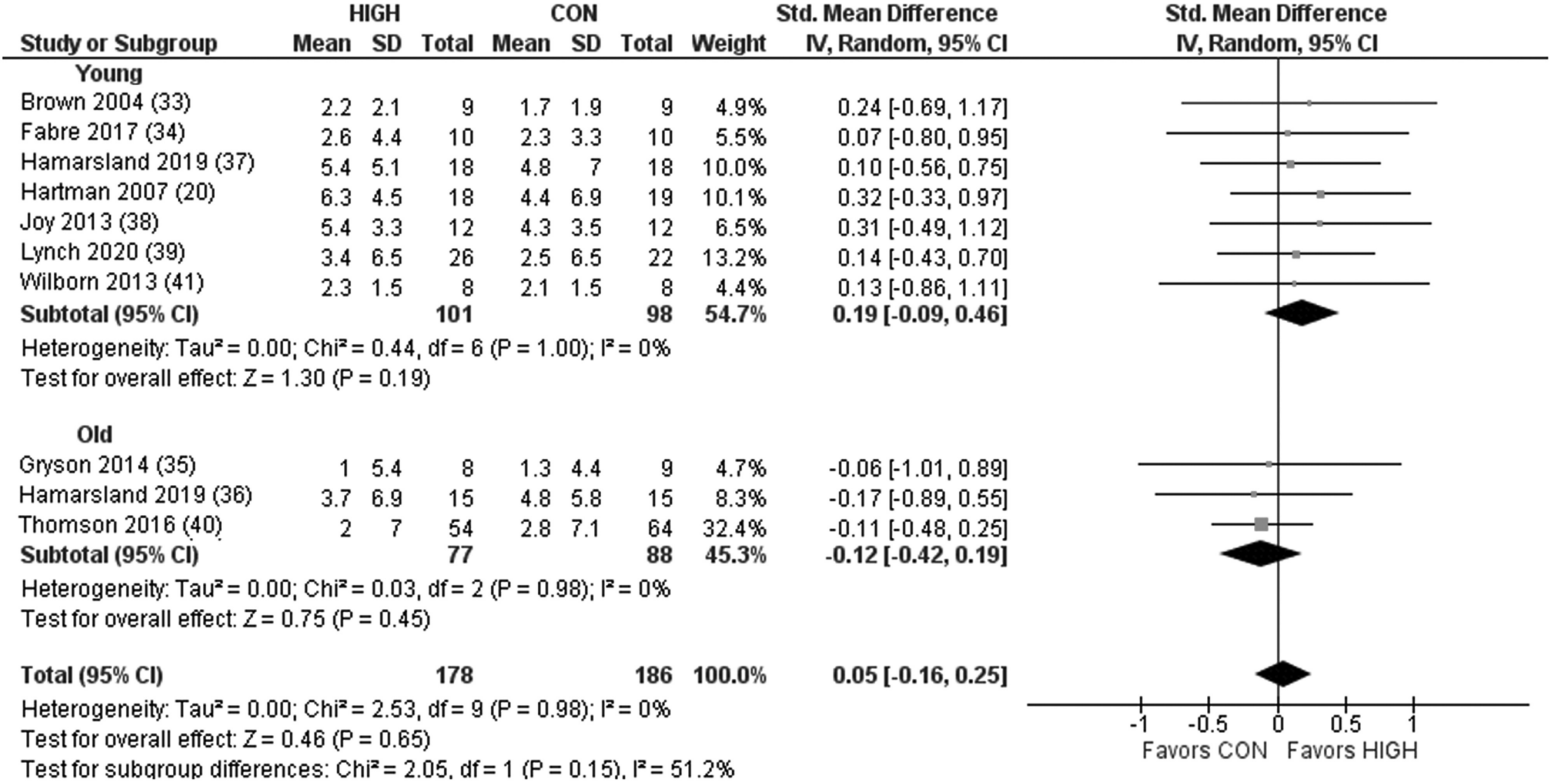
Forest plot of the results from a random-effects meta-analysis shown as the standardized mean difference (SMD) with 95% CIs on indices of lean body mass in young and older participants (model 3). Values on x-axes represent SMD. For each study, the symbol represents the standardized mean *relative* difference (%) of the intervention effect with the horizontal line intersecting it as the lower and upper limits of the 95% CI. The vertical line represents nil effect of either a lower- or higher-quality protein source. The rhombi represent the weighted young, older, and total group's mean difference. The left-hand side favors CON and right-hand side favors HIGH. CON, control protein; HIGH, high-quality protein.

**FIGURE 5:**
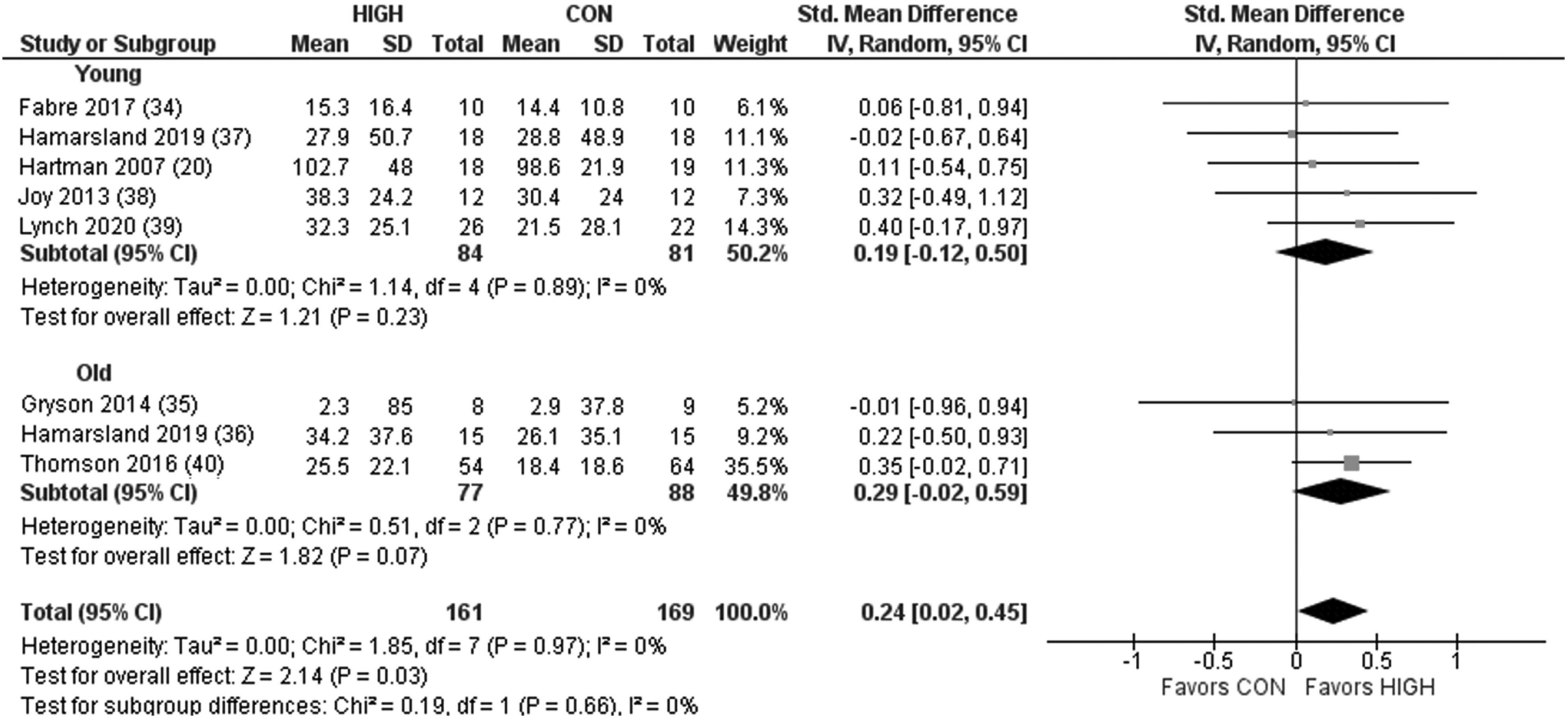
Forest plot of the results from a random-effects meta-analysis shown as standardized mean difference (SMD) with 95% CIs on indices of muscle strength (i.e., 1 repetition maximum; maximal voluntary contraction) in young and older participants (model 3). Values on x-axes represent SMD. For each study, the symbol represents the standardized mean *relative* difference (%) of the intervention effect with the horizontal line intersecting it as the lower and upper limits of the 95% CI. The vertical line represents nil effect of either a lower- or higher-quality protein source. The rhombi represent the weighted young, older, and total group's mean difference. The left-hand side favors CON and right-hand side favors HIGH. CON, control protein; HIGH, high-quality protein.

Protein quality demonstrated a significant effect on postprandial MPS (**Figure 2**). Subgroup analysis revealed a significant effect favoring HIGH in the older but not young adults. For model 2, there was a significant effect of protein quality on RE-induced postprandial MPS favoring HIGH (**Figure 3**). Subgroup analysis revealed a significant effect favoring HIGH in the young and older adults. For model 3, there was no significant effect on LBM across all studies when comparing HIGH with CON, in young or older adults (**Figure 4**). However, although a significant effect was observed favoring HIGH on strength across all studies, no statistically significant effect was observed within the independent groups of young and older adults (**Figure 5**).

### Young compared with older adults

A systematic review of the literature was also conducted on the efficacy of protein quality between age groups, and this can be viewed within **Supplemental Tables 2–4** for models 1–3, respectively. For model 1, the relative change in postprandial MPS across both age groups ranged from −18% to 130% in favor of HIGH (MD: +41 ± 42%). The mean difference in MPS between protein sources was marginally higher in the older compared with the young (42% compared with 31%, respectively) adults, albeit with a large degree of variation and a small sample in the latter. Only 1 study arm, in older participants, was associated with a lower mean stimulation of MPS in HIGH compared with CON (whey: 0.050% ⋅ h^−1^; casein: 0.053% ⋅ h^−1^) (48). One study in young participants also reported a lower mean rate of MPS in HIGH compared with CON (whey: 0.058% ⋅ h^−1^; porcine blood protein: 0.048% ⋅ h^−1^; porcine muscle protein: 0.063% ⋅ h^−1^), but this was not statistically significant (42). For model 2, the relative change in postprandial RE-induced MPS across both age groups ranged from −9% to 117% in favor of HIGH (MD: +33 ± 33%). The mean change between protein sources was higher in older compared with young (38% compared with 29%, respectively) adults. Only 2 study arms from the same study (in young participants) were associated with a lower mean stimulation of MPS in HIGH compared with CON (whey: 0.054% ⋅ h^−1^; casein: 0.059% ⋅ h^−1^; milk: 0.059% ⋅ h^−1^) (46). Another study, in older participants, reported identical means between HIGH and CON (whey: 0.090% ⋅ h^−1^; caseinate: 0.090% ⋅ h^−1^) (47). For model 3, we identified only 3 studies in older compared with 7 in young adults. Combined with the large degree of variability in primary outcome variables typically associated with longitudinal RET intervention studies, no meaningful conclusions could be drawn. However, in the limited evidence available, there was no indication of an age-specific difference in the efficacy of protein quality on longer-term changes to LBM and/or strength.

### Sensitivity analysis

Sensitivity analysis was performed by excluding studies one at a time from each model to evaluate potential outlying studies. However, the removal of any study did not influence the difference in means or significance in postprandial MPS (model 1), RE-induced postprandial MPS (model 2), and LBM and/or strength (model 3). Furthermore, in no instance did a fixed-effect meta-analysis deliver a different magnitude of effect or significance compared with the random-effects meta-analyses employed within the present review. Similarly, use of SMD or MD did not influence the final outcome of any models.

## Discussion

Previous reviews have reached varying conclusions on the efficacy of protein source/quality for indices of skeletal muscle anabolism. Thus, we undertook the first comprehensive and contemporary review to directly examine the efficacy of the quality/source of dietary protein on indices of skeletal muscle anabolism, including postprandial MPS (alone or after a single bout of RE) and muscle adaptations to prolonged (≥6-wks) RET in healthy younger and older adults. Our main findings were that, in agreement with our hypothesis, higher-quality dietary protein supplementation elevated postprandial MPS (model 1) and RE-induced postprandial MPS (model 2) to a greater extent than a dose-matched lower-quality control protein. In addition, higher-quality dietary protein sources augmented longer-term RET-induced increases in muscular strength (model 3). In contrast to our hypothesis, no effect of protein source/quality was observed for changes in LBM with prolonged RET. These observations were consistent between young and older individuals.

### Muscle protein synthesis

Given the favorable EAA profile of complete proteins, it has been suggested that protein source/quality may have an important influence on MPS (22, 65, 66). Herein, we found a small but significant effect of protein source/quality on postprandial MPS (Figure 2) and the RE-induced postprandial MPS response (Figure 3) favoring the higher-quality protein, which equated to an average difference between CON and HIGH of 41% and 33% in models 1 and 2, respectively. It is interesting to note that the clear effect of protein source/quality on postprandial MPS in models 1 (rest) and 2 (combined with exercise) was apparent despite a number of these studies assessing proteins of relatively high quality (i.e., whey compared with casein). Indeed, assessing protein sources that could be defined as being more distinctively different, as assessed via a number of metrics of protein quality, may have produced even more remarkable differences in postprandial MPS. Aside from differences in AA composition and digestibility, the prominent effect of protein source/quality on MPS might also be explained by an artifact of the typical timeframe of MPS assessment (67, 68). However, we could not identify patterns that revealed a superior effect of whey protein across all studies included in this review when the timeframe of assessment of MPS was shorter, and indeed, the average timeframe of assessment for models 1 and 2 was both >4 h. Although beyond the scope of this review, it is pertinent to note that many lower-quality proteins are partly defined by lower rates of digestibility and digestion kinetics, which could explain the inferior muscle anabolic response (48, 69–71).

Although we found a small number of studies whereby the mean difference in postprandial MPS favored lower-quality sources (45, 48), this may be due to this study providing an optimal dose of protein to stimulate MPS or that the comparator proteins (i.e., casein and milk compared with whey) were still of relatively high quality (10). Nevertheless, although the superior muscle anabolic properties of higher, predominantly isolated, quality protein sources were apparent, it is important to note that such conclusions are based on dose-matched protein sources, and it may be possible to ingest larger doses or combinations of lower-quality proteins to elicit a similar MPS response to higher-quality sources (24, 48, 72, 73).

Based on the notion that older individuals require higher per-meal doses of protein to stimulate MPS and that there appears to be a ceiling effect of protein dose for MPS stimulation (6, 9, 10), we also speculated that the effect of protein quality on indices of muscle anabolism may be greater in older compared with younger individuals. However, the effect of protein source/quality was comparable between young and older individuals, albeit not statistically significant in young individuals following feeding only. Interestingly, though, evidence of an age-related muscle anabolic resistance (74) was observed following protein feeding (old: 0.051 ± 0.029% ⋅ h^−1^; young: 0.064 ± 0.017% ⋅ h^−1^) and protein feeding combined with RE (old: 0.061 ± 0.025% ⋅ h^−1^; young: 0.083 ± 0.030% ⋅ h^−1^), with a ∼20% reduction in MPS in older individuals broadly consistent with previous observations (74).

### Lean body mass and strength

It is reasonable to expect that the superior effects of higher-quality sources of protein on postprandial MPS may lead to favorable long-term changes in RET-induced skeletal muscle adaptation (i.e., strength and LBM). However, no significant effect of protein source/quality was observed for the change in LBM with chronic RET (Figure 4) in either young or older individuals. By contrast, a significant effect on strength was observed favoring HIGH (Figure 5), despite no detectable statistically significant effect within the independent groups of young or older adults. This disparity between the acute (models 1 and 2) and chronic (model 3 LBM) findings is not entirely surprising given the reported discord between acute measures of MPS and longer-term skeletal muscle adaptation (75). Specifically, measuring rates of MPS in a strictly controlled laboratory environment may not readily translate to chronic adaptations in free-living conditions, where many other factors beyond acute postprandial MPS stimulation contribute to longer-term RET muscle remodeling (75, 76). Furthermore, it is worthy of note that although fat mass can be accurately and precisely measured with DXA, the measurement of muscle mass is more challenging. Indeed, DXA assesses “lean mass,” which includes not only muscle mass but also soft tissues such as vascular, fibrotic, and connective tissue, as well as organ weight and water (77). Given that all but 1 study in our review used DXA to assess changes in muscle mass, this may explain some of the lack of observations in LBM despite a significant effect on strength. Indeed, appendicular lean mass (as measured via DXA) is commonly used in protein metabolism research as a surrogate measure of skeletal muscle, yet it is only modestly correlated with direct measures of muscle mass (77). Another explanation for the lack of agreement between the MPS outcomes in model 2 and LBM changes in model 3 could be the low sample size available for the meta-analyses for model 3, combined with the well-described interindividual variability in longer-term skeletal muscle remodeling and variability between the methods of muscle mass assessment (78).

In contrast to the lack of effect of RET-induced LBM, our observation that the source/quality of a daily protein supplement across ≥6-wk RET had a discernible effect on strength was remarkable given the multitude of other, arguably more important factors that would influence muscle remodeling responses (i.e., RET program variables, daily protein intake, sleep, genetic predisposition etc.). Although such observations, with the small number of studies available, may be a result of a *false-positive* finding, it may also be worthy of note that improvements to strength (due in part to elevated rates of MPS) are often observed prior to significant changes in LBM, and thus the training studies presented within this review may have not been sufficiently long to detect significant and meaningful differences between interventions. We identified only 1 study within our inclusion criteria that might be considered to have assessed a bona fide lower-quality protein (rice isolate), but this was provided as a 48-g daily bolus (17.4 g EAA), on top of a diet containing adequate daily protein (∼1.9 g · kg^−1^ · day^−1^), which may have minimized any potential effect of protein source/quality on RET outcomes (38). Ultimately, the current consensus on the role of protein source/quality on long-term changes to LBM and strength with RET is inconclusive and warrants further well-designed research studies, particularly longitudinal investigations, with appropriate protein comparators and dietary controls. Studies included in the present analysis focused predominantly on measures of maximal strength, whereas the impact of protein source on performance and functional outcomes in young and older adults remains unclear. This point is important to consider in future studies, particularly given the discord between RET-induced adaptations in strength and functional performance in older adults (79), as well as evidence suggesting that higher-quality dietary protein is associated with functional status (80).

### Considerations

The present review presents a number of considerations and limitations beyond those already discussed. First, in selected cases, defining specific protein sources as higher or lower quality was difficult. For example, Burd et al. (44) investigated the effect of minced beef (containing 13.0 g EAA and 2.5 g leucine) compared with skim milk (containing 13.0 g EAA and 2.7 g leucine). Although we classified skim milk as the higher-quality protein given our quality-defining criteria outlined above, it would be reasonable to suggest that the minced beef may be of equivalent quality based on the nutrient density of this food source. This current review is also subject to the available research to date, which has investigated a limited number of complete protein sources of varying quality with a focus primarily on isolated dairy-source supplements. As most of an individual's dietary protein intake comes from whole-food sources, the relative dearth of such foods as independent variables in the current analysis not only highlights a general limitation of work in this field but also limits the real-world translation and ecological validity of our findings. Indeed, compared with whole-food proteins, isolated proteins are typically devoid of other nutrients and demonstrate more rapid digestion and absorption kinetics, making them ideally suited to acute MPS investigations over a several-hour postprandial timeframe (67). By contrast, whole-food protein sources contain other non-protein-derived nutrients that may affect intramuscular anabolic signaling, MPS, and tissue remodeling (27, 28).

It should also be acknowledged that the assessment of protein source/quality in this review did not differentiate between studies of mixed and isolated myofibrillar MPS. This is important, as the MPS response to protein nutrition may be more accurately detected in the myofibrillar fraction compared with mixed muscle (containing myofibrillar, collagenous, and sarcoplasmic proteins) (31). Our search revealed 4 studies that did not assess myofibrillar MPS in isolation. Although the removal of any single study did not influence the final outcome of our models (assessed via sensitivity analysis), the individual study responses within our analyses may need to be considered against the specific protein fraction assessed. Finally, as aforementioned, it is pertinent to note that we included both crossover and parallel trials in our analyses. In doing so, crossover trials were treated as parallel trials and, therefore, carried less weight. However, the removal of all selected crossover studies did not markedly affect our final outcome measures and/or the size of any potential effect.

Although not a primary purpose of the present analysis, a review of the literature revealed a higher mean difference on postprandial MPS of higher-quality protein sources under conditions of suboptimal (<0.24 g · kg^−1^ for young and <0.4 g · kg^−1^ for old) compared with optimal (+53% and +26%, respectively) protein provision, which opens up an interesting and clinically relevant avenue for future research. Similarly, for model 2, the relative change in RE-induced postprandial MPS was higher with suboptimal (+48%) compared with optimal (+24%) protein doses. Interestingly, analysis indicated that the role of protein source/quality on MPS stimulation may be more pronounced in older individuals at suboptimal doses (model 1: 16% compared with 69%; model 2: 17% compared with 55%, for optimal and suboptimal, respectively), whereas such marked differences between doses were not observed in young individuals. The notion that protein source/quality may be important for MPS stimulation in older adults under suboptimal intakes is supported by data demonstrating that increasing the proportion of leucine in a low dose of EAA enhanced postprandial MPS in older but not younger adults (81). Therefore, it may be prudent for older adults to consider ingesting higher-quality protein to support muscle anabolism, particularly when the per-meal protein dose may be suboptimal for maximal or, indeed, insufficient for robust MPS stimulation. However, more research on whole foods is required to confirm such speculation. Importantly, although many of the studies included in our review are not conclusive on their own, together they present a persuasive pattern for a superior impact of higher-quality proteins on markers of muscle anabolism.

## Conclusions

To our knowledge, we performed the first systematic review and meta-analysis comparing the effect of different, predominantly isolated, protein sources of varying quality on the acute postprandial MPS response at rest and in combination with a resistance exercise bout. We also investigated changes in whole-body skeletal muscle mass and strength following supplementation of different protein sources during prolonged resistance exercise training programs. Protein source/quality demonstrated a significant effect on postprandial MPS both at rest and following resistance exercise. However, although we found a significant effect of protein source/quality on changes in strength with prolonged resistance exercise training, protein source/quality was not associated with favorable changes to lean body mass compared with the lower-quality control proteins. Future research is warranted to assess a more diverse range of protein sources of divergent quality and with whole foods as opposed to isolated protein sources to maximize ecological validity and application.

## Supplementary Material

nxab055_Supplemental_Files

## Data Availability

Data described in the manuscript, code book, and analytic code will be made available upon request pending.

## Notes

This work was not supported by any grants external to the University of Birmingham. Dunhill Medical Trust supported the salary of PTM.

Author disclosures: The authors report no conflicts of interest.

Supplemental Tables 1–4 are available from the “Supplementary data” link in the online posting of the article and from the same link in the online table of contents at https://academic.oup.com/jn/.

Abbreviations used: AA, amino acid; CON, control (quality) protein; EAA, essential amino acid; HIGH, high-quality protein; LBM, lean body mass; MD, mean difference (for meta-analysis); MPS, muscle protein synthesis; RCT, randomized control trials; RE, resistance exercise (i.e., acute bout); RET, resistance exercise training; RM, repetition maximum; SMD, standardized mean difference (for meta-analysis).
