# Supplementary material for: Protein Source and Quality for Skeletal Muscle Anabolism in Young and Older Adults: A Systematic Review and Meta-Analysis
Source: J Nutr. 2021 Apr 13;151(7):1901–20. doi: 10.1093/jn/nxab055 (PMC8245874; doi:10.1093/jn/nxab055)
Supplement: nxab055_Supplemental_Files [file nxab055_supplemental_files.zip › Supplementary table 1.docx]

| **Supplementary table 1.** Summary for Model 1 CON vs. HIGH: the effect of protein source/quality on acute postprandial muscle protein synthesis in young and old adults.^1^ | | | | | | | | |  |
| --- | --- | --- | --- | --- | --- | --- | --- | --- | --- |
|  | Participants,  n^2^ | Age,  years | Protein dose, g | EAA content, g | Leucine content, g | Time period of MPS assessment, hours | Postprandial MPS, %/hour | Relative change MPS, % from CON | |
|  |  |  |  |  |  |  |  |  | |
| TOTAL |  |  |  |  |  |  |  |  | |
| Total CON | 178 | 59 ± 22 | 25.1 ± 8.8 | 9.5 ± 3.9 | 2.0 ± 0.8 | 4.3 ± 1.4 | 0.048 ± 0.022 | - | |
| Total HIGH | 116 | 62 ± 19 | 24.9 ± 9.1 | 12.2 ± 4.4 | 3.0 ± 1.0 | 4.4 ± 1.5 | 0.061 ± 0.033 | 41 ± 42 | |
| *OLD* |  |  |  |  |  |  |  |  | |
| Old CON | 130 | 71 ± 2 | 27.3 ± 8.9 | 10.3 ± 3.9 | 2.2. ± 0.9 | 4.8 ± 1.3 | 0.045 ± 0.023 | - | |
| Old HIGH | 92 | 71 ± 2 | 26.4 ± 9.4 | 13.2 ± 4.2 | 3.2 ± 1.0 | 4.8 ± 1.4 | 0.059 ± 0.035 | 42 ± 42 | |
| *YOUNG* |  |  |  |  |  |  |  |  | |
| Young CON | 48 | 24 ± 1 | 18.5 ± 4.1 | 7.4 ± 3.4 | 1.5 ± 0.5 | 2.8 ± 0.3 | 0.059 ± 0.014 | - | |
| Young HIGH | 24 | 24 ± 1 | 18.2 ± 4.5 | 8.1 ± 2.7 | 2.1 ± 0.3 | 2.8 ± 0.4 | 0.075 ± 0.023 | 31 ± 44 | |
| ^1^Values are mean ± SD. Data not weighted for sample size per study.  ^2^Number of participants across all included studies for each sub-category.  ^3^CON, Control protein; EAA, Essential amino acid; HIGH, High quality protein; MPS, Muscle protein synthesis. | | | | | | | | | |
