# Supplementary material for: Protein Source and Quality for Skeletal Muscle Anabolism in Young and Older Adults: A Systematic Review and Meta-Analysis
Source: J Nutr. 2021 Apr 13;151(7):1901–20. doi: 10.1093/jn/nxab055 (PMC8245874; doi:10.1093/jn/nxab055)
Supplement: nxab055_Supplemental_Files [file nxab055_supplemental_files.zip › Supplementary table 2.docx]

| **Supplementary table 2.** Summary of model 2 CON^1^ vs. HIGH^2^: the effect of protein source/quality on acute postprandial muscle protein synthesis following a bout of resistance exercise in young and old adults.^1^ | | | | | | | | |  |
| --- | --- | --- | --- | --- | --- | --- | --- | --- | --- |
|  | Participants,  n^2^ | Age,  years | Protein dose, g | EAA content, g | Leucine content, g | Time period of MPS assessment, hours | Postprandial MPS, %/hour | Relative change MPS, % from CON | |
|  |  |  |  |  |  |  |  |  | |
| TOTAL |  |  |  |  |  |  |  |  | |
| Total CON | 152 | 44 ± 24 | 23.5 ± 6.9 | 9.7 ± 3.1 | 1.9 ± 0.7 | 4.3 ± 1.4 | 0.064 ± 0.023 | - | |
| Total HIGH | 134 | 27 ± 24 | 23.9 ± 7.4 | 11.7 ± 3.3 | 2.8 ± 0.8 | 4.2 ± 1.4 | 0.083 ± 0.033 | 33 ± 33 | |
| *OLD* |  |  |  |  |  |  |  |  | |
| Old CON | 63 | 71 ± 2 | 26.7 ± 9.0 | 10.5 ± 4.3 | 2.2 ± 0.9 | 4.1 ± 1.2 | 0.052 ± 0.021 | - | |
| Old HIGH | 65 | 70 ± 3 | 26.7 ± 9.0 | 13.4 ± 3.8 | 3.3 ± 0.8 | 4.1 ± 1.2 | 0.071 ± 0.027 | 38 ± 28 | |
| *YOUNG* |  |  |  |  |  |  |  |  | |
| Young CON | 89 | 24 ± 2 | 21.1 ± 3.7 | 9.1 ± 1.9 | 1.8 ± 0.4 | 4.3 ± 1.6 | 0.074 ± 0.021 | - | |
| Young HIGH | 69 | 24 ± 2 | 21.0 ± 4.2 | 10.1 ± 1.6 | 2.4 ± 0.4 | 4.3 ± 1.6 | 0.095 ± 0.037 | 29 ± 38 | |
| ^1^Values are mean ± SD. Data not weighted for sample size per study.  ^2^Number of participants across all included studies for each sub-category.  ^3^CON, Control protein; EAA, Essential amino acid; HIGH, High quality protein; MPS, Muscle protein synthesis. | | | | | | | | | |
