# Supplementary material for: Protein Source and Quality for Skeletal Muscle Anabolism in Young and Older Adults: A Systematic Review and Meta-Analysis
Source: J Nutr. 2021 Apr 13;151(7):1901–20. doi: 10.1093/jn/nxab055 (PMC8245874; doi:10.1093/jn/nxab055)
Supplement: nxab055_Supplemental_Files [file nxab055_supplemental_files.zip › Supplementary table 3.docx]

| **Supplementary table 3**. Summary of model 3 CON vs. HIGH: the effect of protein source/quality when combined with resistance exercise training on longer-term adaptations to lean body mass and strength in young and old adults.^1^ | | | | | | | |
| --- | --- | --- | --- | --- | --- | --- | --- |
|  | Participants,  n^2^ | Age, years | Protein dose, g | EAA content, g | Daily protein intake, g·kg^-1^day^-1^ | ∆ LBM, % from CON | ∆ Strength  % from CON |
|  |  |  |  |  |  |  |  |
| TOTAL |  |  |  |  |  |  |  |
| Total CON | 185 | 36 ± 21 | 32.3 ± 12.0 | 12.0 ± 6.5 | 1.5 ± 0.3 | - | - |
| Total HIGH | 178 | 36 ± 21 | 32.0 ± 12.8 | 15.0 ± 7.2 | 1.5 ± 0.4 | 9 ± 26 | 21 ± 36 |
| *OLD* |  |  |  |  |  |  |  |
| Old CON | 87 | 66 ± 7 | 25.1 ± 14.2 | 9.5 ± 11.0 | 1.3 ± 0.2 | - | - |
| Old HIGH | 87 | 65 ± 7 | 25.7 ± 15.0 | 12.8 ± 10.7 | 1.3 ± 0.2 | -25 ± 4 | 10 ± 51 |
| *YOUNG* |  |  |  |  |  |  |  |
| Young CON | 98 | 24 ± 3 | 35.5 ± 10.5 | 12.7 ± 5.7 | 1.6 ± 0.4 | - | - |
| Young HIGH | 101 | 24 ± 4 | 34.7 ± 11.9 | 15.6 ± 6.9 | 1.6 ± 0.4 | 23 ± 13 | 27 ± 31 |
| ^1^Values are mean ± SD. Data not weighted for sample size per study.  ^2^Number of participants across all included studies for each sub-category.  ^3^CON, Control protein; EAA, Essential amino acid; HIGH, High quality protein; LBM, Lean body mass. | | | | | | | |
