# Supplementary material for: Protein Source and Quality for Skeletal Muscle Anabolism in Young and Older Adults: A Systematic Review and Meta-Analysis
Source: J Nutr. 2021 Apr 13;151(7):1901–20. doi: 10.1093/jn/nxab055 (PMC8245874; doi:10.1093/jn/nxab055)
Supplement: nxab055_Supplemental_Files [file nxab055_supplemental_files.zip › Supplementary table 4 (search strategy).docx]

| **Supplementary table 4.** Search strategy to identify studies assessing the impacts of protein source/quality on markers of skeletal muscle anabolism^1^. | |
| --- | --- |
| 1 | Muscle protein synth* |
| 2 | Muscle protein synthesis |
| 3 | MPS |
| 4 | Fractional synth* |
| 5 | Fractional synthetic rate |
| 6 | FSR |
| 7 | Phenylalanine |
| 8 | Postprandial |
| 9 | Protein quality |
| 10 | Protein |
| 11 | Essential amino acids |
| 12 | EAA |
| 13 | Essential amino |
| 14 | DIASS |
| 15 | PDCAAS |
| 16 | Milk |
| 17 | Whey |
| 18 | Casein |
| 19 | Soy |
| 20 | Rice |
| 21 | Wheat |
| 22 | Pea |
| 23 | Egg |
| 24 | Hypertrophy |
| 25 | Strength |
| 26 | Training |
| 27 | Resistance |
| 28 | Exercise |
| ^1^The search was limited by identifying human studies only. Boolean operators ‘and’ and ‘or’ were used to combine all listed search terms. | |
